# Quantitatively estimating main soil water-soluble salt ions content based on Visible-near infrared wavelength selected using GC, SR and VIP

**DOI:** 10.7717/peerj.6310

**Published:** 2019-01-22

**Authors:** Haifeng Wang, Yinwen Chen, Zhitao Zhang, Haorui Chen, Xianwen Li, Mingxiu Wang, Hongyang Chai

**Affiliations:** 1 Key Laboratory of Agricultural Soil and Water Engineering in Arid and Semiarid Areas, Ministry of Education, Northwest A&F University, Yangling, Shaanxi, China; 2 College of Water Resources and Architectural Engineering, Northwest A&F University, Yangling, Shaanxi, China; 3 Department of Foreign Languages, Northwest A&F University, Yangling, Shaanxi, China; 4 Department of Irrigation and Drainage, China Institute of Water Resources and Hydropower Research, Beijing, China; 5 Department of Civil and Environmental Engineering, University of California, Irvine, CA, USA

**Keywords:** Soil salinization, Water-soluble salt ions, VIS-NIR, GC, SR, VIP, Model

## Abstract

Soil salinization is the primary obstacle to the sustainable development of agriculture and eco-environment in arid regions. The accurate inversion of the major water-soluble salt ions in the soil using visible-near infrared (VIS-NIR) spectroscopy technique can enhance the effectiveness of saline soil management. However, the accuracy of spectral models of soil salt ions turns out to be affected by high dimensionality and noise information of spectral data. This study aims to improve the model accuracy by optimizing the spectral models based on the exploration of the sensitive spectral intervals of different salt ions. To this end, 120 soil samples were collected from Shahaoqu Irrigation Area in Inner Mongolia, China. After determining the raw reflectance spectrum and content of salt ions in the lab, the spectral data were pre-treated by standard normal variable (SNV). Subsequently the sensitive spectral intervals of each ion were selected using methods of gray correlation (GC), stepwise regression (SR) and variable importance in projection (VIP). Finally, the performance of both models of partial least squares regression (PLSR) and support vector regression (SVR) was investigated on the basis of the sensitive spectral intervals. The results indicated that the model accuracy based on the sensitive spectral intervals selected using different analytical methods turned out to be different: VIP was the highest, SR came next and GC was the lowest. The optimal inversion models of different ions were different. In general, both PLSR and SVR had achieved satisfactory model accuracy, but PLSR outperformed SVR in the forecasting effects. Great difference existed among the optimal inversion accuracy of different ions: the predicative accuracy of Ca^2+^, Na^+^, Cl^−^, Mg^2+^ and SO_4_^2−^ was very high, that of CO_3_^2−^ was high and K^+^ was relatively lower, but HCO_3_^−^ failed to have any predicative power. These findings provide a new approach for the optimization of the spectral model of water-soluble salt ions and improvement of its predicative precision.

## Introduction

Soil salinization, one of the most important causes of land desertification and deterioration, has posed serious threat to agricultural development and sustainable utilization of natural resources (Shahid & Rahman, 2011; Abbas et al., 2013). 950 million ha of soil worldwide has become salinized (Schofield & Kirkby, 2003). Soil salinization is eroding and degenerating the arable soil at the speed of 10 ha/min (Graciela & Alfred, 2009). Soil remediation and management are very difficult in China because of such complex natural factors as climate, terrain and geology, and human factors as unreasonable irrigation and disruption of ecological balance. The total area of saline soil in China is 36 million ha (Li et al., 2014), accounting for 4.88% of the total area available nationwide (The National Soil Survey Office, 1998). Saline soil usually has a high concentration of salt ions with a series of effects on the plants such as physiological draught, ion toxicity and metabolic disorder, thus forming “salt damage” (Munns, 2002; Tavakkoli et al., 2011). In addition, one major cause of the inaccuracy of soil salinity spectral measurement is that pure salts seldom exist in the soil because of some trace salt ion elements are always fixed in soil crystals. Therefore, quick and accurate acquisition of the detailed information of the various salt ions content in the soil can enhance the pertinence and effectiveness of saline soil management.

The traditional quantitative estimation of soil salt contents usually includes such steps as field soil sampling in fixed points, experiments in the laboratory and comprehensive statistical analysis (Urdanoz & Aragüés, 2011). Such a method is incapable of the dynamic monitoring of saline soil in a large area because of its high consumption of time and energy, small number of measuring points and poor representativeness (Ding & Yu, 2014). Compared with conventional laboratory analysis methods, remote sensing technology has been widely used due to its rich information, continuity, high precision and low cost (Ben-Dor, 2002; Viscarra Rossel et al., 2006; Viscarra Rossel & Behrens, 2010; Viscarra Rossel & Webster, 2012). The various soil constituents (contents of water, salt, organic matter and so forth) can be acquired conveniently from remote sensing data (Gomez, Viscarra Rossel & McBratney, 2008; Yu et al., 2010; Periasamy & Shanmugam, 2017). Hence, with the abundant spectral reflection information within the VIS-NIR intervals of soil salinity, it is feasible to improve the accuracy of soil salinization inversion (Al-Khaier, 2003; Ben-Dor et al., 2009; Abbas et al., 2013).

The application of VIS-NIR spectral analysis technique has been proved effective in improving the accuracy of quantitative estimation and eliminating the external disturbance to some extent (Dehaan & Taylor, 2002; Metternicht & Zinck, 2003; Farifteh et al., 2008). The univariate linear regression on the basis of soil salinity index developed for CR (continuum removed) reflectance can be used as a method for soil salt content estimation (Weng, Gong & Zhu, 2008). Due to the strong correlation between soil electrical conductivity (EC) and soil salinity, EC is also one of the important indicators for evaluating soil salinization degree. A variety of approaches have been used to acquire the EC in the field soil, including the partial least squares regression (PLSR) and multivariate adaptive regression splines (MARS) (Volkan Bilgili et al., 2010; Nawar, Buddenbaum & Hill, 2015), logarithmic model (Xiao, Li & Feng, 2016a), Bootstrap-BP neural network model (Wang et al., 2018d) and satellite remote sensing technology (Nawar et al., 2014; Bannari et al., 2018). In addition, the differential transformation (Xia et al., 2017) and fractional derivative (Wang et al., 2017; Wang et al., 2018c) can fully utilize the potential spectral information and enhance model accuracy. The methods of spectral classification (Jin et al., 2015) and water influence elimination (Chen et al., 2016; Peng et al., 2016b; Yang & Yu, 2017) work well in improving the quantitative inversion accuracy of soil salinity. Therefore, the remote sensing technique is reliable to inverse the soil salinity quantitatively on different scales.

The quantitative analysis of VIS-NIR spectral intervals can help evaluate the content of some chemical elements (Viscarra Rossel et al., 2006; Farifteh et al., 2008; Cécillon et al., 2009; Ji et al., 2016) due to the different characteristic absorption spectrum in soil chemical elements. Besides, there exists a correlation between some principal salt ions (Na^+^, Cl^−^) and spectral reflectance (Jiang et al., 2017). Therefore, VIS-NIR spectroscopy technique can be used to obtain the contents of the soil salt ions to a certain extent. The spectral response characteristics of mid-infrared (MIR) spectroscopy are better than those of VIS-NIR spectroscopy in predicting soil salinity information, the latter has high predicting accuracy of the total salts content, HCO_3_^−^, SO_4_^2−^ and Ca^2+^, followed by Mg^2+^, Cl^−^ and Na^+^ (Peng et al., 2016a). The spectral models have satisfactory prediction of the SAR (sodium absorption ratio) of soil salinization evaluation parameter, which is composed of the contents of Ca^2+^, Mg^2+^ and Na^+^ (Xiao, Li & Feng, 2016b). Qu et al. (2009) found that the contents of the total salt, SO_4_^2−^, pH and K^+^+Na^+^ have a higher inversion accuracy using spectral data to create PLSR model. The different pretreatment of the different ion models varies by creating and analyzing PLSR model that demonstrates relatively good predictive effects like ion contents of Ca^2+^, Mg^2+^, SO_4_^2−^, Cl^−^, and HCO_3_^−^ (Dai et al., 2015). Overall, PLSR is a frequently used and robust linear model for quantitative research because it has inference capabilities which are useful to model a probable linear relationship between the reflectance spectra and the salt ions content in soil. However, the non-uniform data and non-linear reflectance in spectral information of some soil chemical elements lead to the reduction in model accuracy (Viscarra Rossel & Behrens, 2010; Nawar, Buddenbaum & Hill, 2015). In particular, support vector regressions (SVR) based on kernel-based learning methods has the ability to handle nonlinear analysis case with high model accuracy (Vapnik, 1995; Peng et al., 2016a; Hong et al., 2018b). Over the past several decades, the use of SVR for classification and regression has been extensively applied in soil VIS-NIR spectroscopy (Ben-Dor, 2002; Xiao, Li & Feng, 2016b; Hong et al., 2018a). Moreover, the SVR model works well in estimating the contents of K^+^, Na^+^, Ca^2+^ and SO_4_^2−^ in the soil (Wang et al., 2018a). Thus, the correct way of modeling helps to guarantee the model accuracy (Farifteh et al., 2007).

Many researches focused on the inversion of soil salinity using spectral information. Nevertheless, little research has explored the eight water-soluble salt ions (K^+^, Ca^2+^, Na^+^, Mg^2+^, Cl^−^, SO_4_^2−^, HCO_3_^−^ and CO_3_^2−^) using spectral information in the soil. The model fitting of ions and spectral information still needs improving (Farifteh et al., 2008; Peng et al., 2016a). Apart from the suitable multivariate statistical analysis method that can partly improve the inversion effects, reduction of redundant information is another identified approach to further optimize the model (Bannari et al., 2018; Stenberg et al., 2010). Plenty of studies have demonstrated that spectral variable selection methods can not only reduce the complexity of calibration models, but also improve the model predictive performance (Hong et al., 2018a). To select the optimal spectral variable subset, scholars have investigated varied methods such as gray correlation (GC) (Li et al., 2016; Wang et al., 2018b), stepwise regression (SR) (Zhang et al., 2018) and variable importance in projection (VIP) (Qi et al., 2017), and have achieved satisfactory effects. In addition, all the three methods have been widely applied in many studies, such as plant physiology, food engineering, mathematical statistics (Oussama et al., 2012; Maimaitiyiming et al., 2017; Liu, Yang & Wu, 2015). However, few studies have concentrated on the use of variable selection algorithms in the inversion of soil salt ions.

This study aims to: (1) build the optimal model of soil salt ions using VIS–NIR spectroscopy technique; (2) compare the models based on the sensitive spectral ranges selected using GC, SR and VIP methods for different soil ions; (3) compare the performance of PLSR and SVR models, and identify the optimal models for different ions.

## Materials and Methods

### Study area

Hetao Irrigation District (HID), with Yin Mountains at its north, the Yellow River at its south, Ulanbuh Desert at its west and Baotou at its east, lies in Bayannur League, Inner Mongolia, China. It consists of irrigation areas of Ulan Buh, Jiefangzha, Yongji, Yichang and Urat, and it is China’s largest irrigation district with a total size of 5740 km^2^ (Yu et al., 2010). In addition, HID is an important production base of cereal and oil plants in China with major crops of wheat, corn and sunflower. Shahaoqu Irrigation Area (SIA), a typical region of saline soil in HID, was chosen as the study area. SIA (107°05′∼107°10′E, 40°52′∼41°00′N) is located in the central east of Jiefangzha Irrigation Area. SIA belongs to typical continental climate, having hot summers, chilly winters, rare precipitation and strong evaporation. Its mean annual temperature, precipitation, potential evaporation is about 7.1 °C, 155 mm and 2,000 mm, respectively. Physiographically, the mean elevation and slope of SIA are about 1,030 m and 1/10,000, respectively. According to the World Reference Base for Soil Resources (WRB), the local soil texture is mainly silty clay loam with varying degrees of saline soil. Over the years, due to its gentle terrain slope, poor groundwater runoff, intense land surface evaporation and irrational farming activities, about 60% of the land within the district has been affected by various degree of salinization, which seriously restricted the agricultural development (Wu et al., 2008; Gao et al., 2015).

### Sample collection and chemical analysis

**Figure 1:**
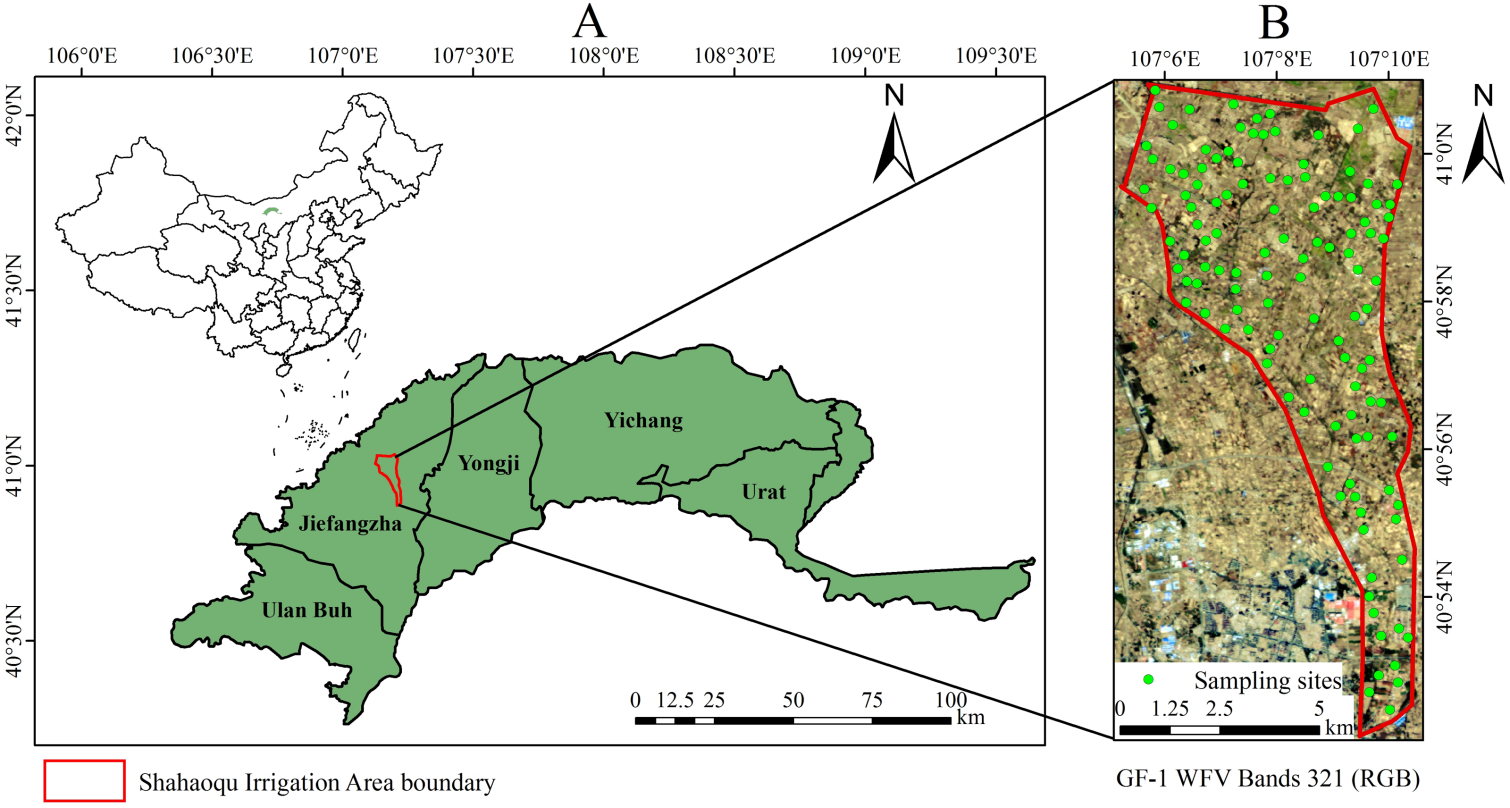
Distribution of sampling sites in the study area. (A) Location map of Shahaoqu Irrigation Area. (B) Sampling location in Shahaoqu Irrigation Area.

The Hetao irrigation district administration gave field permit approval to us (NO. 2017YFC0403302). To ensure the representativeness of soil samples, the samples were randomly gathered from a total of 120 sampling units on a grid of 16 m ×16 m (because the spatial resolution of GF-1 satellite imagery is 16 m) in the study area during October 12∼22, 2017 (Fig. 1). In each unit, approximately 0.5 kg of topsoil (0–5 cm) was collected at four randomly selected sampling sites and then mixed thoroughly to obtain a representative sample. Overall, a total of 120 soil samples were acquired, and each sample was stored in a plastic bag, labeled and sealed. A portable global position system (GPS) was used to determine the coordinates of sampling points. Subsequently, the soil samples were transported to the lab to receive a series of such treatments as sufficient natural air-drying for two weeks and rubbing through a 2 mm sieve to exclude small stones and other impurities. Each sample was divided into two subsamples to be used for spectra collection and physiochemical analysis.

Each 50 g of soil sample was put into a respective flask, and 250 ml of distilled water (the ratio of water to soil is 5:1) were added into each flask. The water-soluble ion contents were measured in the filtrate obtained from full soaking, oscillation and filtration (Aboukila & Norton, 2017). Ca^2+^ and Mg^2+^ were measured using EDTA titration, Na^+^ and K^+^ flame photometry, CO_3_^2−^ and HCO_3_^−^ double indicator-neutralization titration, Cl^−^ silver nitrate titration, and SO_4_^2−^ EDTA indirect complexometry (Bao, 2000). The content of CO_3_^2−^ was too low (approximately 0) in some soil samples because CO_3_^2−^ is liable to integrate with Ca^2+^ and Mg^2+^ as sediment in a weak alkaline solution (Table 1). Coefficient of variation (CV) reflects the degree of discreteness, and a positive correlation exists in two variables. The high CV helps to build a robust model (Dai et al., 2015). The grading of CV showed a wide range of variation among different ions, among which the ion contents of K^+^, Na^+^ and SO_4_^2−^ are over 100%, showing a strong variability, and those of CO_3_^2−^, Cl^−^, Ca^2+^, Mg^2+^ and HCO_3_^−^ are between 10% and 100%, having a moderate variability.

**Table 1:** Descriptive statistics of soil water-soluble salt ions content.

Statistical index	Minimum/ g kg^−1^	Maximum/ g kg^−1^	Mean/ g kg^−1^	Standard deviation	Coefficient of variation/%
CO_3_^2−^	0.000	0.066	0.020	0.020	98.86
HCO_3_^−^	0.171	0.666	0.316	0.099	31.27
SO_4_^2−^	0.047	40.892	9.073	10.828	119.34
Cl^−^	0.145	23.234	4.825	4.711	97.65
Ca^2+^	0.080	4.111	0.697	0.669	95.95
Mg^2+^	0.039	1.952	0.706	0.606	85.91
K^+^	0.001	5.727	0.936	1.358	145.14
Na^+^	0.016	23.035	5.014	5.563	110.94

### Laboratory spectral measurements and pretreatments

The soil samples were put into black vessels with a diameter of 10 cm and depth of 2 cm for spectral data collection and the surfaces were smoothed with a straightedge in the laboratory. The spectral data of the soil samples were measured using ASD (Analytical Spectral Devices, Inc., Boulder, CO, USA) FieldSpec^®^3 spectrometer with spectral range from 350–2,500 nm. This instrument is equipped with two sensors whose spectral resolutions are 1.4 nm and 2 nm, for the region of 350–1,000 nm and 1,000–2,500 nm, respectively. The spectral data was measured in a dark room with the light sources which have halogen lamps of 50 W, 50 cm from the sample soil surfaces, and 30° incident angle to reduce the effects of external factors to the minimum. The field angle of fiber-optics probe is 5°, and it is 15 cm from the sample soil surface. The light source and spectrometer had been fully preheated, and the spectrometer had been corrected with a standardized white panel (99% reflectance) prior to each measurement to reduce measurement error. Each sample soil was measured in four directions (3 turns, each is 90°), the spectrum was collected five times in each direction, and altogether there were 20 curves of the spectrum (Hong et al., 2018b). These curves were used as the raw spectral reflectance (*R*_raw_) after having the arithmetic mean in ViewSpecPro software version 6.0. The gaps of the spectral curves near 1,000 nm and 1,800 nm were corrected using the Splice Correction function (Xiao, Li & Feng, 2016a).

**Figure 2:**
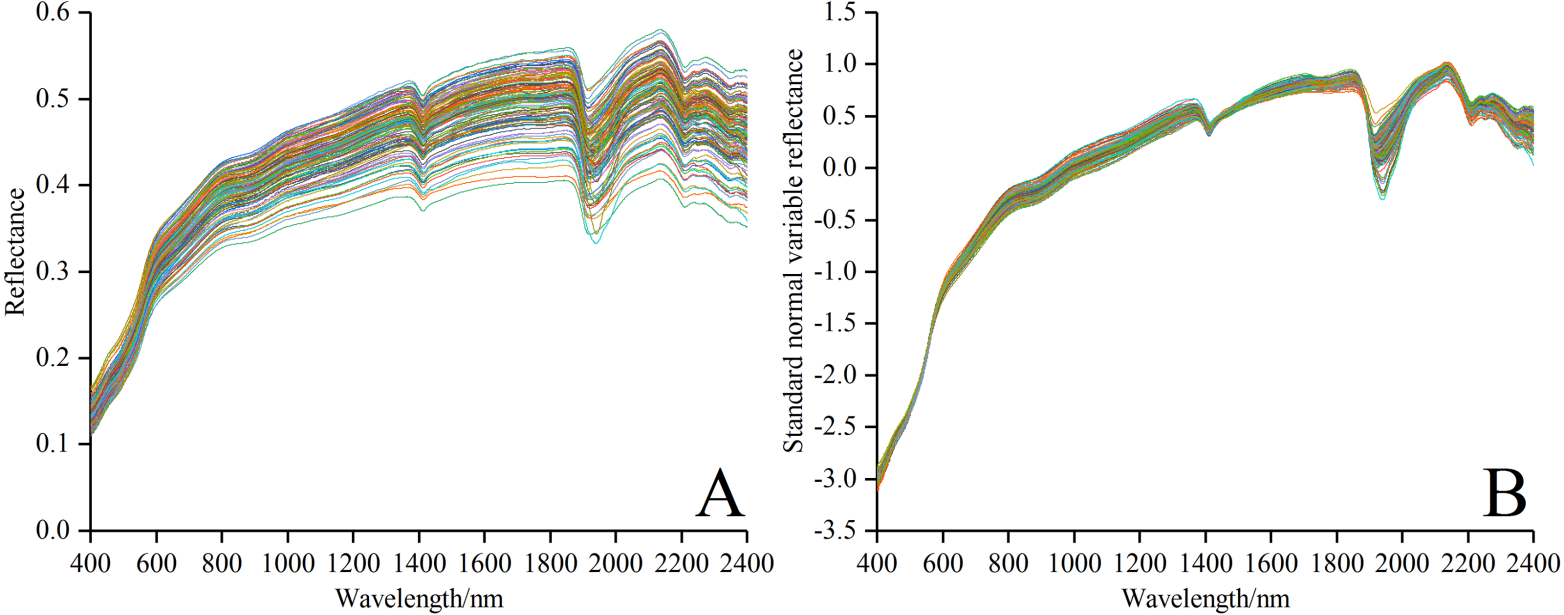
Spectral curves of all soil samples. (A) Reflectance spectral curves. (B) Standard normal variable reflectance curves.

The fluctuation would affect the accuracy of subsequent modeling because of such disturbance as the external environment, instrument noise and random error in spectral data collection. In general, a series of effective pretreatment, including smoothing, resampling and transformation etc., can eliminate the external noise to some degree, and then enhance the spectral characteristics (Ding et al., 2018). Therefore, it is necessary to pretreat *R*_raw_ in the following steps. (i) The marginal wavelength (350–399 nm and 2,401–2,500 nm) of higher noise in each soil sample was removed, then remaining spectrum data was smoothed with filter method (window size is 5 and polynomial order is 2) using Savitzky-Golay (SG) (Savitzky & Golay, 1964) via Origin Pro software version 2017SR2. (ii) The spectral data between 400 and 2,400 nm was resampled with a 10 nm of sample interval to keep the spectral features and remove redundant information (Xu et al., 2016). A new spectral curve consisting of 200 wave bands was obtained. (iii) The precise *R*_raw−SNV_ was obtained by using the standard normal variable (SNV) to eliminate the effects of soil particle size, surface scattering and baseline shift on the spectrum data (Xiao, Li & Feng, 2016b; Barnes, Dhanoa & Lister, 1989). The spectral curves of *R*_raw_ and *R*_raw−SNV_ are shown in Figs. 2A and 2B. Notably, comparison indicated that the spectral curve in Fig. 2B was much smoother than that in Fig. 2A, which made for the subsequent modeling.

### Gray correlation (GC)

The GC, as one grey system theory, seeks the primary and secondary relations and analyzes the different effects of all the factors in a system (Deng, 1982; Li et al., 2016). Its calculation process is as follows: the reference sequence is ${X}_{0}= \left\{ {x}_{0} \left( t \right) ,t=1,2,\ldots ,n \right\} $, the comparative sequence is ${X}_{i}= \left\{ {x}_{i} \left( t \right) ,t=1,2,\ldots ,n \right\} $, and the formula of the gray correlation degree (GCD) between *X*_0_ and *X*_*i*_ is

(1) \begin{eqnarray*}\mathrm{GCD}= \frac{1}{n} \sum _{t=1}^{n}\gamma \left( {x}_{0} \left( t \right) ,{x}_{i} \left( t \right) \right) \end{eqnarray*}

where $\gamma \left( {x}_{0} \left( t \right) ,{x}_{i} \left( t \right) \right) = \frac{{\min }_{i}{\min }_{t} \left\vert {x}_{0}(t)-{x}_{i}(t) \right\vert +\rho {\max }_{i}{\max }_{t} \left\vert {x}_{0}(t)-{x}_{i}(t) \right\vert }{ \left\vert {x}_{0}(t)-{x}_{i}(t) \right\vert +\rho {\max }_{i}{\max }_{t} \left\vert {x}_{0}(t)-{x}_{i}(t) \right\vert } $

*ρ* is the distinguishing coefficient within $ \left[ 0,1 \right] $ . *ρ* was set as 0.1 in this paper.

The inconsistent dimension between the spectral data and the contents of different ions has some effects on the data analysis. Therefore, normalizing the spectral data preprocessing method can reduce these disadvantageous effects (Liu, Yang & Wu, 2015; Wang et al., 2018b). In this paper, the larger the GCD of a certain band is, the closer relation the band and the ion content has, and vice versa.

### Variable importance in projection (VIP)

The VIP is a variable selection method based on PLSR (Oussama et al., 2012). The explanatory power of the independent variables to the dependent variables is achieved by calculating the VIP score. The independent variables are sequenced according to the explanatory power (Qi et al., 2017). The VIP score for the *j*-th variable is given as:

(2) \begin{eqnarray*}{\mathrm{V IP}}_{j}=\sqrt{ \frac{p\ast \sum _{f=1}^{F}{\mathrm{SSY }}_{f}\ast {\mathrm{W}}_{jf}^{2}}{{\mathrm{SSY }}_{total}\ast F} }\end{eqnarray*}

Where *p* is the number of independent variables; *f* is the total number of components; SSY_*f*_ is the sum of squares of explained variance for the *f*-th component and *p* the number of independent variables. SSY_*total*_ is the total sum of squares explained of the dependent variable. ${\mathrm{W}}_{jf}^{2}$ gives the importance of the *j*-th variable in each *f*-th component. The higher value VIP_*j*_ has, the stronger explanatory power the independent variable has over the dependent variable. The VIP scores of independent variables have been recognized as a useful measure to identify important wavelengths when the score is more than 1 (Wold, Sjöström & Eriksson, 2001; Maimaitiyiming et al., 2017).

### Model construction and validation

Two-thirds of the samples were used for modeling (*n* = 80) and one third for validation (*n* = 40) using Kennard-Stone (K-S) to calculate the Euclidean distance among different samples to ensure the statistical characteristics of modeling and the validation datasets resembled that of the whole sample set (Kennard & Stone, 1969).

The PLSR and SVR models were applied to the quantitative inversion of different water-soluble salt ion contents in the saline soil in this paper. The PLSR model is a new stoichiometric statistical model. Compared with the traditional multivariate least squares regression (MLSR), PLSR can overcome the multicollinearity among the variables, reduce the dimension, synthesize and filter the information, extract the aggregate variables with the strongest explanatory power in the system, and exclude the noise with no explanatory power (Wold, Sjöström & Eriksson, 2001). The optimal fitting model was built using the number of optimal principal components through full cross validation. SVR model is a new machine learning method based on the principle of structural risk minimization provided by the statistical learning theory. This model is characterized by its ability of solving such problems as limited sample size, nonlinear data processing and spatial pattern recognition of high-dimension data (Vapnik, 1995). During the modeling in this study, the type of SVR and kernel were set as epsilon-SVR and linear function, respectively; the penalty parameter *C* and nuclear parameter *g* were acquired by a grid-searching technique and a leave-one-out cross validation procedure. The optimal values of *C* and *g* were selected when the minimum RMSE_CV_ (root mean squared error of cross validation) was produced (Xiao, Li & Feng, 2016b). The two models were constructed and validated using the Unscrambler software version X10.4 (CAMO AS Oslo, Oslo, Norway).

Precision indices of determination coefficient of calibration (*R*_*c*_^2^), determination coefficient of prediction (*R*_*p*_^2^), root mean squared error (RMSE) and ratio of performance to deviation (RPD) were used to evaluate the performance of these models. RPD classification was adopted to facilitate the interpretation of predictive results: a model is considered as excellent when RPD ≥ 2.5, as very good when 2.0 ≤ RPD < 2.5, as good when 1.8 ≤ RPD <2.0, and as satisfactory when 1.4 ≤ RPD <1.8 and can only distinguish between high and low values when 1.0 ≤ RPD <1.4 (Viscarra Rossel, Taylor & McBratney, 2007). Generally, the most robust model would be the one with the largest *R*_*c*_^2^, *R*_*p*_^2^ (approach to 1) and RPD value and the lowest RMSE value.

## Results

### Correlation between water-soluble salt ions content and spectral reflectance

**Figure 3:**
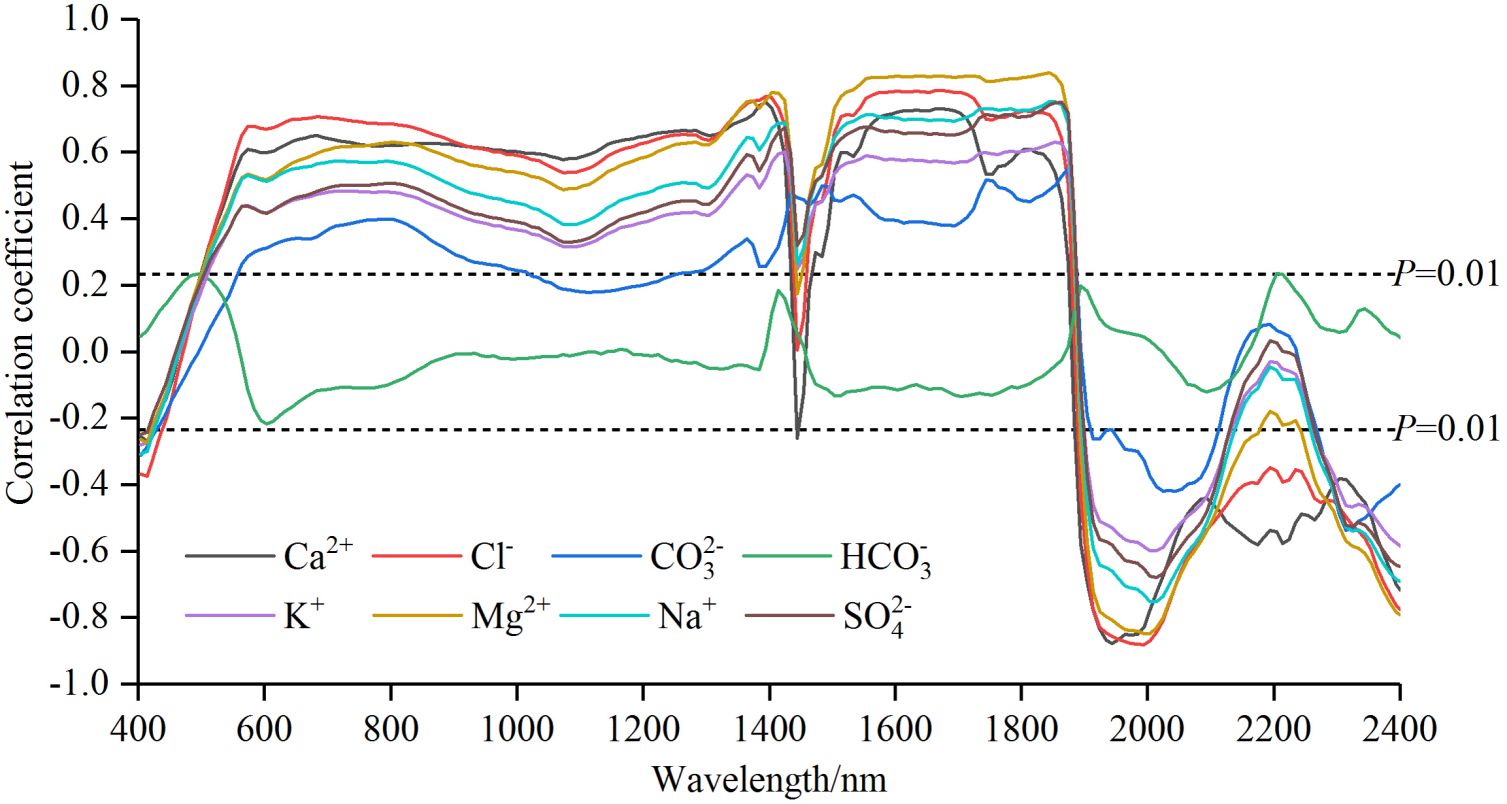
Correlation coefficients of soil water-soluble salt ions content with standard normal variable reflectance.

The correlation coefficients (Pearson correlation) between each soil salt ion content and *R*_raw−SNV_ in the range of 400–2,400 nm were tested with the significance level of *P* < 0.01 (|*r*| = 0.234 or above). The curves of correlation coefficients of soil salt ions were plotted in Fig. 3 and the numbers of bands passing the significance test were counted in Table 2.

**Table 2:** Max correlation coefficient and band intervals of soil water-soluble salt ions content with standard normal variable reflectance.

Water-soluble salt ions	Number of significant bands	Maximum correlation coefficient	Maximum correlation band intervals/nm
Ca^2+^	190	−0.877	1,940∼1,950
Cl^−^	192	−0.882	1,990∼2,000
CO_3_^2−^	146	0.552	1,870∼1,880
HCO_3_^−^	1	0.235	2,200∼2,210
K^+^	178	0.630	1,850∼1,860
Mg^2+^	186	−0.848	1,990∼2,000
Na^+^	181	−0.752	2,010∼2,020
SO_4_^2−^	178	0.749	1,860∼1,870

The curve patterns of SO_4_^2−^, Cl^−^, Ca^2+^, Mg^2+^, K^+^ and Na^+^ were similar (Fig. 3). From 400 nm to about 550 nm, the correlation coefficients rose sharply from negative to positive, moved with a gentle depression until 1,400 nm, plummeted and surged up to 1,560 nm (among the curves, the change of Ca^2+^ was the sharpest), and maintained a relative stable state to 1850 nm. And then from 1,850 to 2,400 nm, dramatic oscillating variations alternated between rise and fall. In the intervals of 400–1,400 nm and 1,850–2,400 nm the curve pattern of CO_3_^2−^ was similar to that of other ions such as SO_4_^2−^. But between 1,400 nm and about 1,850 nm, the curve took on a unique pattern: sustained oscillating rise. The coefficient curve of HCO${}_{3}^{-}$ displayed a smaller variation, smoothly fluctuating between −0.2 and 0.2. The complex variation of the coefficient curves of different ions revealed rich spectral information.

**Figure 4:**
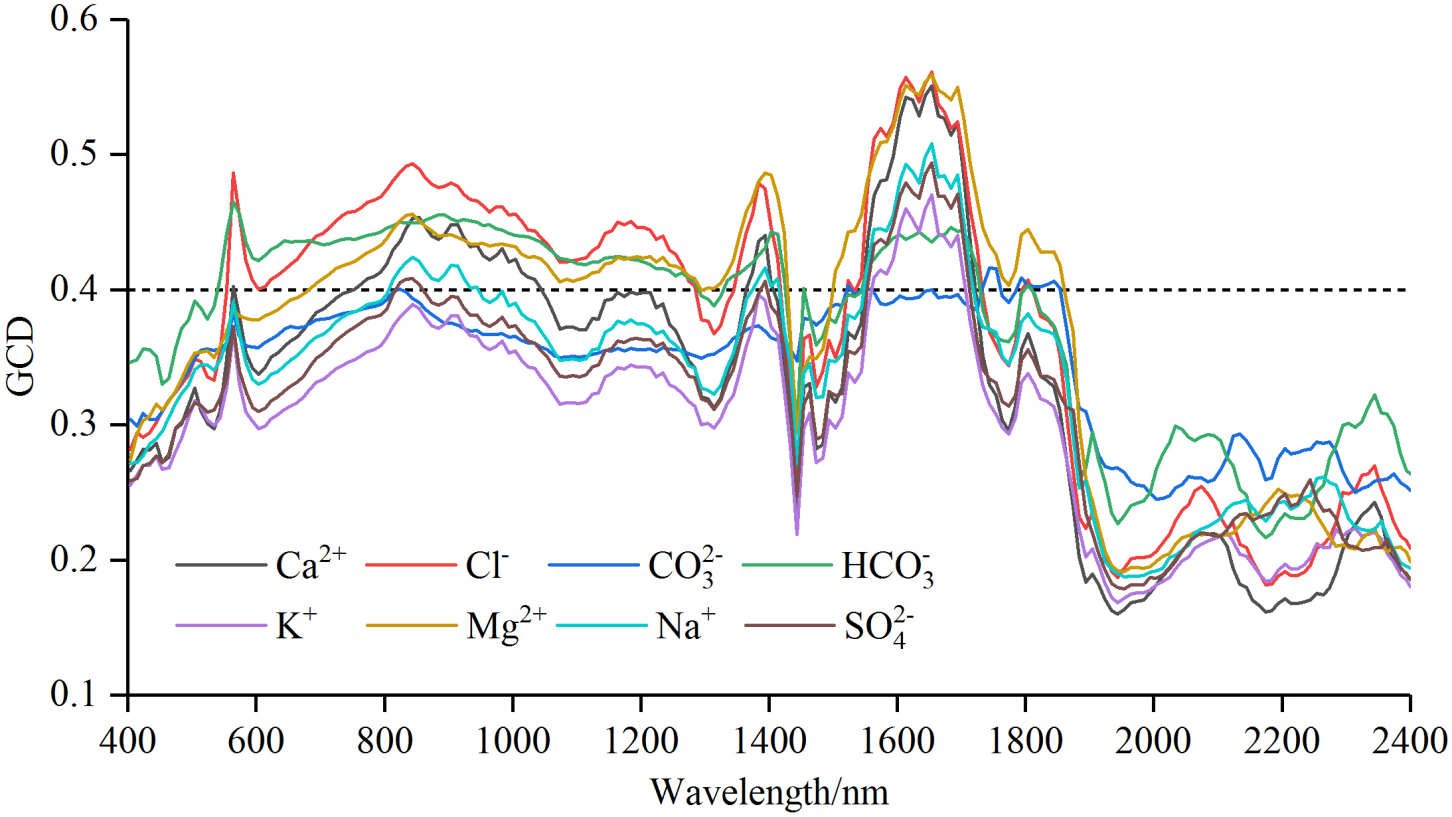
Gray correlation degree (GCD) for soil water-soluble salt ions content with standard normal variable reflectance.

### Selection of characteristic wavelength

#### Characteristic wavelength selection based on GC method

The curves of gray correlation degree for soil water-soluble salt ions content and *R*_raw−SNV_ were shown in Fig. 4. The correlation coefficient curves of the seven ions except CO_3_^2−^ resembled those of the GCD of the *R*_raw−SNV_. Generally, the curves exhibited patterns of “oscillatory rise, fluctuation, rapid rise and fall, and oscillatory fluctuation”. The gray correlation curves of CO_3_^2−^ followed a pattern of “ascending, plummeting, and smooth transition”. The analysis of the GC curve amplitude showed the amplitudes of Cl^−^, Mg^2+^ and Ca^2+^ were relatively large, and those of Na^+^, SO_4_^2−^, K^+^ and HCO_3_^−^ were relatively small, and that of CO_3_^2−^ was relatively gentle.

The order of the maximal GCD was: Cl^−^ (0.561) > Mg^2+^ (0.559) > Ca^2+^ (0.551) > Na^+^ (0.508) > SO_4_^2−^ (0.494) > K^+^ (0.470) > HCO_3_^−^ (0.465) > CO_3_^2−^ (0.416). To ensure that each salt ion had sensitive bands as far as possible, the GCD threshold value was set as 0.40 to select the wavelength. The sensitive band was counted through gray correlation method (Table 3). The numbers of sensitive bands of different ions could be sequenced from the largest to the smallest as follows: Mg^2+^ (110) > HCO_3_^−^ (105) > Cl^−^ (101) > Ca^2+^ (53) > Na^+^ (36) > SO_4_^2−^ (21) > K^+^ (15) > CO_3_^2−^ (14). Therefore, the orders of sensitive band numbers and maximal GCD values had great difference. Furthermore, the band intervals corresponding to the maximum GCD of different salt ions were as follows: CO_3_^2−^ was near-infrared between 1,740 and 1,750 nm, HCO_3_^−^ was green light between 560 and 570 nm, and the rest of six ions were near-infrared between 1,650 and 1,660 nm.

**Table 3:** Max gray correlation degree and band intervals of soil water-soluble salt ions content with standard normal variable reflectance.

Water-soluble salt ions	Sensitive band numbers	Maximum gray correlation degree	Maximum gray correlation degree intervals/nm
Ca^2+^	53	0.551	1,650∼1,660
Cl^−^	101	0.561	1,650∼1,660
CO_3_^2−^	14	0.416	1,740∼1,750
HCO_3_^−^	105	0.465	560∼570
K^+^	15	0.470	1,650∼1,660
Mg^2+^	110	0.559	1,650∼1,660
Na^+^	36	0.508	1,650∼1,660
SO_4_^2−^	21	0.494	1,650∼1,660

#### Characteristic wavelength selection based on SR method

Feature band intervals were selected by stepwise regression method in SPSS software version 23.0 (IBM, Chicago, IL, USA), and the significance levels of variables acceptance and rejection were set at 0.10 and 0.15 (Zhang et al., 2018). The parameter indexes of feature band intervals selection were shown in Table 4 by stepwise regression method at maximum adjusted *R*^2^.

**Figure 5:**
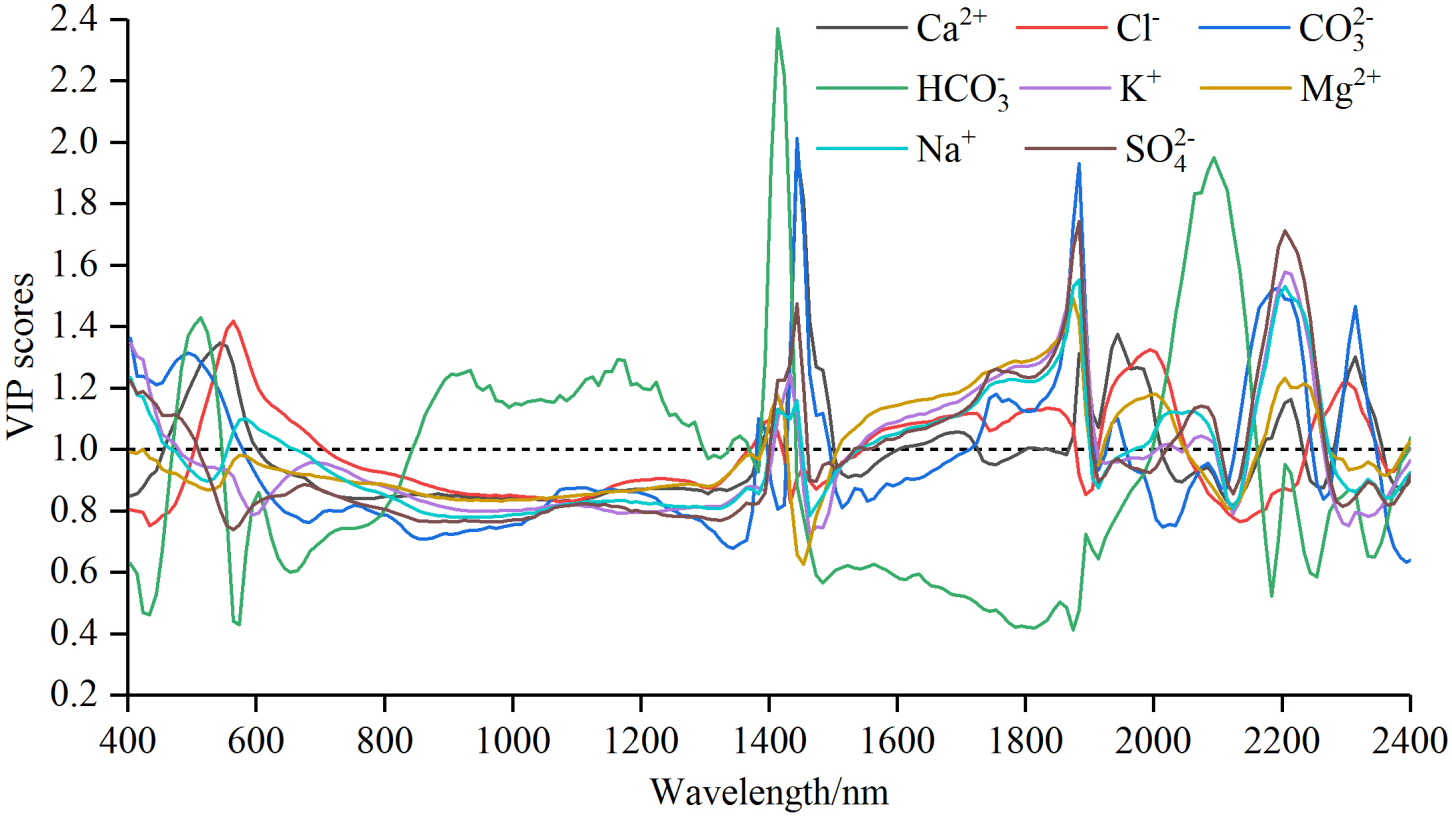
The Variable importance in projection (VIP) scores for soil water-soluble salt ions content with standard normal variable reflectance.

**Table 4:** Parameter indexes of feature band intervals selection by stepwise regression method.

Water-soluble salt ions	Sensitive band numbers	Band intervals/nm	Adjusted *R*^2^	Standard error	Sig.
Ca^2+^	7	1,040∼1,050, 1,090∼1,100, 1,900∼1,910, 1,920∼1,930, 2,200∼2,210, 2,310∼2,320, 2,370∼2,380	0.942	0.529	<0.001
Cl^−^	8	730∼740, 910∼920, 1,890∼1,900, 1,970∼1,980, 1,990∼2,000, 2,180∼2,190, 2,200∼2,210, 2,290∼2,300	0.975	1.063	<0.001
CO_3_^2−^	4	1,280∼1,290, 1,360∼1,370, 1,380∼1,390, 1,420∼1,430	0.836	0.012	<0.001
HCO_3_^−^	3	2,200∼2,210, 2,260∼2,270, 2,290∼2,300	0.934	0.085	<0.001
K^+^	6	740∼750, 810∼820, 1,160∼1,170, 1,890∼1,900, 2,210∼2,220, 2,390∼2,400	0.817	0.706	<0.001
Mg^2+^	6	1,130∼1,140, 1,930∼1,950, 1,990∼2,000, 2,100∼2,110, 2,170∼2,180	0.973	0.152	<0.001
Na^+^	6	740∼750, 820∼830, 1,860∼1,870, 2,210∼2,220, 2,260∼2,270, 2,390∼2,400	0.942	1.812	<0.001
SO_4_^2−^	6	610∼620, 1,140∼1,150, 1,960∼1,970, 2,210∼2,220, 2,290∼2,300, 2,390∼2,400	0.947	3.255	<0.001

Great difference existed among the optimal SR models of different ions, and the numbers of band intervals accepted by the model range from 3 to 8 (Table 4). The SR model fitted well with the adjusted *R*^2^ greater than 0.8 when the number of selected independent variables was considered. Meanwhile, SR model of each ion was statistically significant (*p* < 0.001). Therefore, the band intervals selected by the SR models were used as the independent variables of PLSR and SVR models.

**Table 5:** Max VIP scores and band intervals of soil water-soluble salt ions content with standard normal variable reflectance.

Water-soluble salt ions	Sensitive band numbers	Maximum VIP scores	Maximum VIP scores intervals/nm
Ca^2+^	69	1.97	1,440∼1,450
Cl^−^	85	1.42	560∼570
CO_3_^2−^	67	2.01	1,440∼1,450
HCO_3_^−^	79	2.37	1,410∼1,420
K^+^	69	1.73	1,880∼1,890
Mg^2+^	69	1.49	1,870∼1,880
Na^+^	83	1.55	1,880∼1,890
SO_4_^2−^	74	1.74	1,880∼1,890

#### Characteristic wavelength selection based on VIP method

Curves of VIP scores of soil water-soluble salt ions content and *R*_raw−SNV_ were shown in Fig. 5. Max VIP scores and band intervals obtained from VIP method of soil water-soluble salt ions content and *R*_raw−SNV_ were shown in Table 5.

The curves patterns of seven ions were similar except HCO_3_^−^ (Fig. 5). These curves exhibited violent oscillation in the intervals of 400–800 nm and 1,900–2,400 nm, gentle transition between 800 nm and around 1,400 nm, and fluctuant rise from 1,400 to 1,900 nm. In contrast, the curve of HCO_3_^−^ showed oscillatory rise from 400 to 1,400 nm, a “U” shaped motion from 1,400 to 1,900 nm or so, and a rapid fall and oscillation to 2,400 nm. The numbers of sensitive bands based on VIP method displayed the following sequence: Cl^−^ (85) > Na^+^ (83) > HCO_3_^−^ (79) > SO_4_^2−^ (74) > Mg^2+^ (69) = Ca^2+^ (69) = K^+^ (69) > CO_3_^2−^ (67). The sequence of the maximal VIP scores was HCO_3_^−^ (2.37) > CO_3_^2−^ (2.01) > Ca^2+^ (1.97) > SO_4_^2−^ (1.74) > K^+^ (1.73) > Na^+^ (1.55) > Mg^2+^ (1.49) > Cl^−^ (1.42). The spectral interval of the maximal VIP scores of Cl^−^ was from 560 to 570 nm, Ca^2+^, CO_3_^2−^ and HCO_3_^−^ were concentrated between 1,410 and 1,450 nm; and K^+^, Mg^2+^, Na^+^ and SO_4_^2−^ were from 1,870 to 1,890 nm.

### Construction and analysis of PLSR model

The sensitive bands were obtained using different band selection methods of GC, SR and VIP to build PLSR model. The results of PLSR model were shown in Table 6.

**Table 6:** Calibration and validation results of soil water-soluble salt ions content from the PLSR inversion models using the GC, SR and VIP wavelength selection methods.

Wavelength selection methods	Water-soluble salt ions	Latent variables	Calibration sets	Validation sets		
			*R*_*c*_^2^	*R*_*p*_^2^	RMSE/(g kg^−1^)	RPD
Gray correlation	Ca^2+^	7	0.897	0.724	0.362	1.71
	Cl^−^	7	0.796	0.565	3.150	1.35
	CO_3_^2−^	5	0.660	0.649	0.012	1.21
	HCO_3_^−^	7	0.646	0.285	0.088	0.96
	K^+^	1	0.388	0.258	1.209	0.85
	Mg^2+^	6	0.891	0.767	0.295	1.99
	Na^+^	7	0.840	0.805	2.589	1.88
	SO_4_^2−^	4	0.561	0.360	8.711	0.87
Stepwise regression	Ca^2+^	7	0.965	0.937	0.168	3.95
	Cl^−^	2	0.861	0.729	2.434	1.80
	CO_3_^2−^	4	0.685	0.742	0.010	1.80
	HCO_3_^−^	3	0.340	0.154	0.094	0.64
	K^+^	5	0.722	0.563	0.931	1.37
	Mg^2+^	4	0.933	0.849	0.236	2.52
	Na^+^	3	0.901	0.868	2.145	2.67
	SO_4_^2−^	5	0.918	0.889	3.807	2.75
Variable importance in projection	Ca^2+^	3	0.909	0.865	0.249	2.57
	Cl^−^	4	0.930	0.862	1.725	2.48
	CO_3_^2−^	9	0.865	0.617	0.012	1.44
	HCO_3_^−^	9	0.704	0.263	0.090	0.93
	K^+^	5	0.664	0.566	0.945	1.43
	Mg^2+^	3	0.910	0.840	0.243	2.34
	Na^+^	8	0.939	0.902	1.801	3.15
	SO_4_^2−^	8	0.919	0.872	4.038	2.75

The models of the six ions Ca^2+^, Cl^−^, CO_3_^2−^, Mg^2+^, Na^+^ and SO_4_^2−^ performed well using VIP method (*R*_*c*_^2^ is close to 1). The models based on the bands of Ca^2+^, Cl^−^, Mg^2+^, Na^+^ and SO_4_^2−^ selected using the SR method displayed good fitting effect, and those of Ca^2+^, Mg^2+^ and Na^+^ using the GC method exhibited good fitting effect.

In terms of verification accuracy, VIP method had excellent prediction of Ca^2+^, Na^+^, SO_4_^2−^, SR method had excellent prediction of Ca^2+^, Mg^2+^, Na^+^, SO_4_^2−^ (the RPD of Ca^2+^ was up to 3.95), and GC method did not show strong prediction power over any ions. On the contrary, all the three models demonstrated poor forecasting power over HCO_3_^−^. The RPDs of SR-HCO_3_^−^ and VIP-HCO_3_^−^ were 0.64 and 0.93 respectively. Therefore, VIP method had the best modeling effect and SR method had the best forecasting effect, and GC method had poor modeling and forecasting effects on the salt ions inversion in the PLSR model.

### Construction and analysis of SVR model

The sensitive bands were obtained by using different band selection methods of GC, SR and VIP to build SVR model. The results of SVR model were shown in Table 7.

**Table 7:** Calibration and validation results of soil water-soluble salt ions content from the SVR inversion models using the GC, SR and VIP wavelength selection methods.

Wavelength selection methods	Water-soluble salt ions	Calibration sets	Validation sets		
		*R*_*c*_^2^		*R*_*p*_^2^	RMSE/(g kg^−1^)	RPD
Gray correlation	Ca^2+^	0.910		0.752	0.337	1.73
	Cl^−^	0.652		0.500	3.275	1.05
	CO_3_^2−^	0.688		0.664	0.012	1.14
	HCO_3_^−^	0.563		0.328	0.083	0.70
	K^+^	0.421		0.269	1.155	0.61
	Mg^2+^	0.934		0.781	0.289	2.07
	Na^+^	0.809		0.764	2.851	1.85
	SO_4_^2−^	0.565		0.397	9.046	0.52
Stepwise regression	Ca^2+^	0.964		0.940	0.164	3.97
	Cl^−^	0.893		0.790	2.186	2.15
	CO_3_^2−^	0.605		0.583	0.013	1.16
	HCO_3_^−^	0.327		0.164	0.095	0.56
	K^+^	0.717		0.578	0.874	1.26
	Mg^2+^	0.936		0.875	0.214	2.75
	Na^+^	0.903		0.864	2.171	2.61
	SO_4_^2−^	0.915		0.893	3.862	2.71
Variable importance in projection	Ca^2+^	0.960		0.935	0.173	3.93
	Cl^−^	0.949		0.897	1.483	2.98
	CO_3_^2−^	0.883		0.664	0.012	1.56
	HCO_3_^−^	0.669		0.280	0.088	0.91
	K^+^	0.645		0.565	0.888	1.23
	Mg^2+^	0.965		0.877	0.214	2.51
	Na^+^	0.958		0.872	2.211	2.76
	SO_4_^2−^	0.914		0.865	4.106	2.48

The modeling accuracy of SVR model was similar to that of PLSR model. But the verification accuracy of ions was different between the two models. VIP method had the excellent prediction of Ca^2+^, Cl^−^, Mg^2+^, Na^+^, SR method had the excellent prediction of Ca^2+^, Mg ^2+^, Na^+^, SO_4_^2−^, and GC method did not show strong prediction power over any ions. The prediction results of Ca^2+^ were the best: the RPD of VIP and SR models were 3.93 and 3.97, respectively. Overall, in the SVR model, VIP method exhibited the best performance for modeling and predicting the salt ions content, SR method was the second, and GC method was relatively poorer.

## Discussion

### Comparison among the results of different salt ions content in estimating

**Figure 6:**
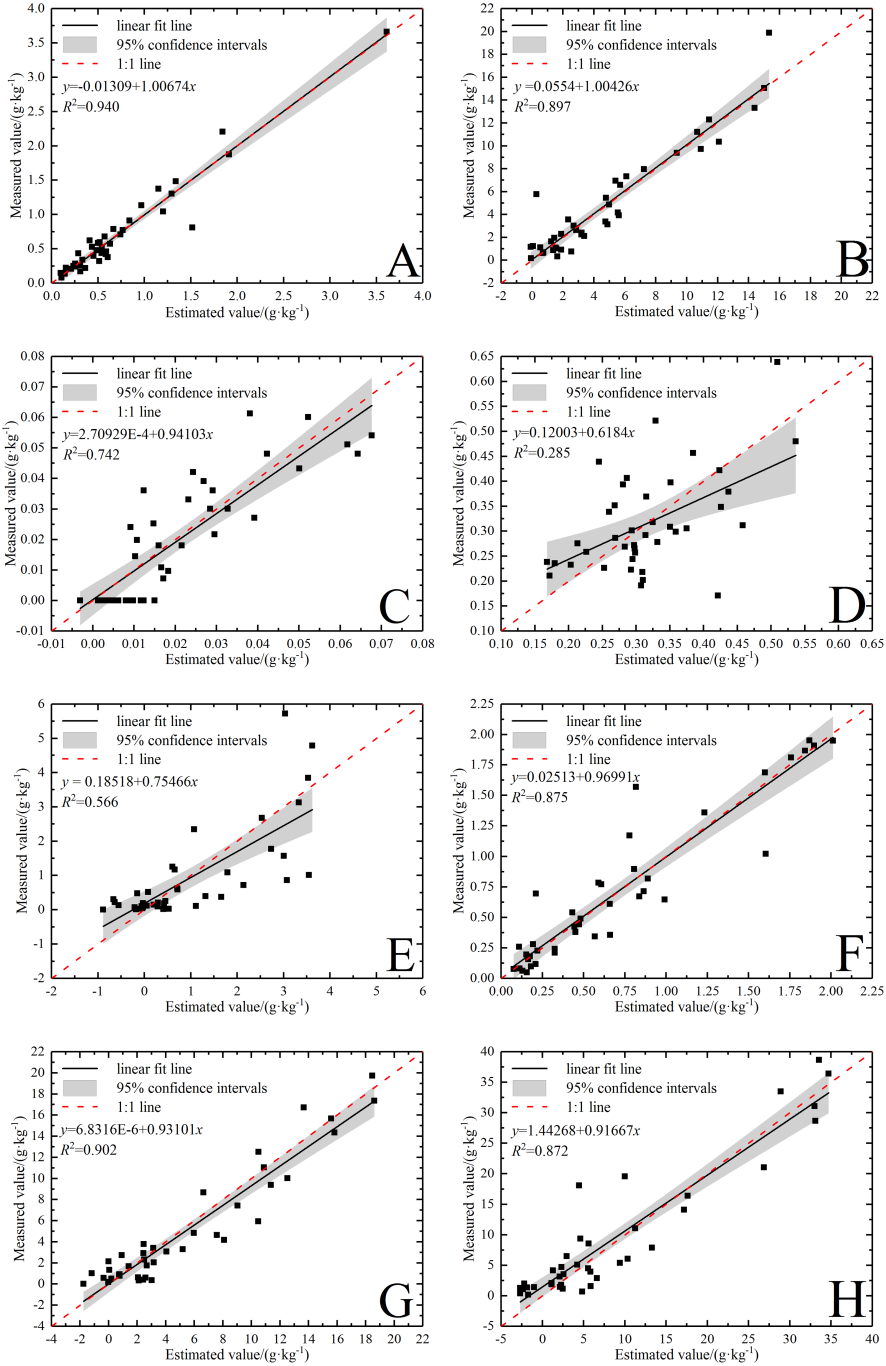
Validation of soil water-soluble salt ions content based on the best model. (A) Ca^2+^ with SR-SVR model. (B) Cl^−^ with VIP-SVR model. (C) CO${}_{3}^{2-}$ with SR-PLSR model. (D) HCO${}_{3}^{-}$ with GC-PLSR model. (E) K^+^ with VIP-PLSR model. (F) Mg^2+^ with SR-SVR model. (G) Na^+^ with VIP-PLSR model. (H) SO${}_{4}^{2-}$ with VIP-PLSR model.

The optimal band selection method varied in some degree from the optimal modeling method (Tables 6 and 7). The comparison was made between the measured value and the estimated value of all the ions concerned under the optimal model (Fig. 6). The sequence of the forecasting power of the ions was Ca^2+^ > Na^+^ > Cl^−^ > Mg^2+^ > SO_4_^2−^ > CO_3_^2−^ > K^+^ > HCO_3_^−^, and it was the same as that of the modeling power.

Obviously, the verification result showed that most data points of the five ions, Ca^2+^, Na^+^, Cl^−^, Mg ^2+^ and SO_4_^2−^, were concentrated near line 1:1. The optimal models of these five ions had very strong predicative power with the RPD above 2.5 (Tables 6 and 7). Compared with the previous researches, model prediction effects of K^+^ and Na^+^ (Qu et al., 2009); Ca^2+^, Na^+^ and Mg^2+^ (Viscarra Rossel & Webster, 2012); HCO_3_^−^, Ca^2+^, Cl^−^, Mg^2+^ and SO_4_^2−^(Dai et al., 2015); HCO_3_^−^, Ca^2+^ and SO_4_^2−^ (Peng et al., 2016a); K^+^, Na^+^, Ca^2+^ and SO_4_^2−^ (Wang et al., 2018a) were satisfactory. Although the results of this study are not exactly the same as these previous researches, it still shows the rationality own to some extent. In addition, this result shows that band selection has realized the goal of removing the irrelevant information, and plays a major role in improving the inversion accuracy of salt ions.

In Fig. 6, the data points of CO_3_^2−^ and K^+^ were relatively dispersed in the verification result. The CO_3_^2−^ had a relatively good predictive power (RPD = 1.80) and the K^+^ had a normal predictive power (RPD = 1.43). Notably, HCO_3_^−^ had no predicative power (RPD = 0.96) because the slope was under the 1:1 line and the data points were most discrete (Fig. 6D). The predicting effect of HCO_3_^−^ was different from that of Peng et al. (2016a) and Dai et al. (2015), but similar to that of Wang et al. (2018a). The cause of this result needs to be further studied. Overall, it is vital to make some efforts to improve the robustness and accuracy of these ion models. Xiao, Li & Feng (2016b) failed to predict Na^+^, Mg ^2+^ and Ca^2+^, but applied the SVR model to forecasting SAR after the SNV transformation and the performance was satisfactory (RPD = 2.13). Analogously, first derivative reflectance (FDR) index was calculated to effectively predict SAR by Xiao, Li & Feng (2016a). In addition, Viscarra Rossel & Webster (2012) forecasted the content of Na^+^ after logarithmic pretreatment with VIS-NIR spectral technique (RPD = 2.10). Thus, salt ion indexes construction and variable transformation processing are helpful approaches to improve the correlation with the spectra so as to establish satisfactory models.

A little difference existed in the applicability between PLSR and SVR models on inversing the content of ions. Both methods could produce satisfactory results in conformity with that of Peng et al. (2016a). In addition, the optimal inversion models and prediction models for each ion were different: SR-PLSR model and SR-SVR model for Ca^2+^, VIP-SVR model and SR-PLSR model for CO_3_^2−^, SR-PLSR model and VIP-PLSR model for K^+^, VIP-PLSR model and GC-PLSR model for HCO_3_^−^, respectively. Among them, the performance of the optimal inversion model of Ca^2+^ resembled that of the prediction model. The results suggested that the ion models with poorer performance frequently demonstrated uncertainty in the inversion process (Peng et al., 2016a). Generally, as the major water-soluble ion components in the two highly soluble salts of sodium and kali, Na^+^ and K^+^ exhibit great difference in the spectral characterization degree (Dai et al., 2015). Therefore, the spectral characters of water-soluble salt ions are not necessarily determined by the number of dissociative ions, so more pertinent experiments and analysis should be conducted to explore the response mechanism.

### Correlation analysis and inversion performance

The raw spectral reflectance curve of each soil sample presented distinct shapes (Fig. 2A). One of the prime reasons for this phenomenon is that the absorption features in these soil samples were related to soil salt crystal contents and types, as well as various chemical bonds (e.g., C-H, O-H, N-H). The results were in accordance with those in previous studies (Viscarra Rossel et al., 2006; Viscarra Rossel & Webster, 2012; Dai et al., 2015; Peng et al., 2016a; Wang et al., 2018a), which demonstrated that soil VIS-NIR spectra could be used to determine part of soil salt ions contents in some degree.

Traditionally, correlation analysis helps reveal the relationships between soil salt ions content and VIS-NIR spectra, and it indicates modeling effects to some degree (Weng, Gong & Zhu, 2008). In the current research, the number of the significant bands of different ions could be sequenced from the largest to the smallest as follows: Cl^−^ (96%) > Ca^2+^ (95%) > Mg^2+^ (93%) > Na^+^ (90.5%) > K^+^ (89%) = SO_4_^2−^ (89%) > CO_3_^2−^ (73%) > HCO_3_^−^ (0.5%), the correlation coefficients of different ions ranged from the largest to the smallest as: Cl^−^ (−0.882) > Ca^2+^ (−0.877) > Mg^2+^ (−0.848) > Na^+^ (−0.752) > SO_4_^2−^ (0.749) > K^+^ (0.630) > CO_3_^2−^ (0.552) > HCO_3_^−^ (0.235) (Table 2). Thereby, five ions (Cl^−^, Ca^2+^, Mg^2+^, Na^+^ and SO_4_^2−^) had more significant relationship with reflectance spectra. Although there were some differences between forecasting power ranking and correlation ranking, the optimal models of these five ions had the excellent predictive results (Fig. 6). Nevertheless, the other three ions (K^+^, CO_3_^2−^ and HCO_3_^−^) had weak correlations and unsatisfactory predictive power. In particular, HCO_3_^−^ had only one significant band and the worst prediction effects. But in most cases, the sensitive band numbers of HCO_3_^−^ were not the least in comparing the results of the three wavelength selection methods (Tables 3–5). Thus, we conjecture that the different calculation mechanisms cause a certain inconsistency between modeling performance and sensitivity. In addition, the optimal method of finding out their responding spectrum varies from one ion to another in the soil. In future study, it is practically significant to adopt various methods to select the optimal bands in the inversion of soil ions.

### Effects of wavelength selection on estimation models

The massive complex spectra often contain a large amount of redundant information irrelevant to the ions contents. The selection of feature spectra is hence a critical step to create a robust model. From Tables 3–5, we could see the great difference exist in the number of wavelength selected with the three methods: VIP method had the largest number of wavelengths (34.5%∼42.5%), SR method had the smallest number of wavelengths (1.5%∼4%) and number of wavelengths (7%∼55%) varied greatly by GC method.

Our experiment with three wavelength selection methods also indicated that different methods yielded different results. Among the three methods, the VIP method produced the best results, followed by SR method, while the GC method performed least ideally. We argue that the GC method is not necessarily an inappropriate method as some results are still acceptable. However, GC method could distinguish the primary relationships among the factors in the system by calculating and comparing GCD (Deng, 1982; Liu, Yang & Wu, 2015). In the field of spectral analysis, the application of GC method could better identify sensitive spectral indices, select sensitive bands and optimize inversion model (Li et al., 2016). On the other hand, Wang et al. (2018b) used GC method to extract the feature bands of soil organic matter content to construct the model with stronger generalization capability. Therefore, the soil compositions have a strong impact on the performance of spectral model. This conclusion is consistent with previous research results (Viscarra Rossel et al., 2006; Viscarra Rossel & Webster, 2012; Xiao, Li & Feng, 2016b). The VIP values were calculated with VIP method, in the process of PLSR analysis to further evaluate the significance of each wavelength for model prediction (Wold, Sjöström & Eriksson, 2001; Maimaitiyiming et al., 2017; Qi et al., 2017). VIP method often produces the best results in the modeling set because it can distinguish between useful information and inevitable noises in the set. Oussama et al. (2012) adopted this method to reduce almost 75% of the total data set for a simplified model of high accuracy. Additionally, as a simplified regression linear model, SR method not only preserves significant bands but also solves multicollinearity problems effectively (Xiao, Li & Feng, 2016a; Xiao, Li & Feng, 2016b). It has great optimization effect on model complexity by adjusting the significance level of selected and excluded variables (Zhang et al., 2018). Compared with the selection results with VIP method, SR method could be used to extract fewer bands to establish ions (except for K^+^, CO_3_^2−^ and HCO_3_^−^) forecasting models with RPD above 1.80. Therefore, it is meaningful to make further simplification of the model while ensuring its accuracy.

### Research limitations

This study clearly demonstrated that VIS-NIR spectral analysis technique is an effective method to detect salt ions content of salinity soil in the irrigated district. In terms of extracting feature wavelengths to estimate ions content, our work provides a comprehensive comparison and evaluation approaches. Such endeavor is critically and practically important to further enhance the model performance of the soil salt ions. The application of machine learning algorithms with strong applicability to solve nonlinear relationship between variables, such as Ant Colony Optimization-interval Partial Least Square (ACO-iPLS), Recursive Feature Elimination based on Support Vector Machine (RF-SVM), and Random Forest (RF) has been proved to be a useful approach to obtain the effective information of soil organic matter (Ding et al., 2018). To further improve the prediction accuracy, the more machine learning algorithms should be applied to the analysis of sensitive spectral regions and the construction of stable models in future study. In addition, the application of multi-source remote sensing platforms such as Landsat, GaoFen-5, Hyperion and unmanned aerial vehicle (UAV) in soil salt ions estimation has not been investigated. Therefore, further research should focus on the possible combination of multiple approaches and remote sensing data at different scales to estimate soil salt ions content.

## Conclusions

This study investigated the feasibility of estimating soil water-soluble salt ions content via VIS-NIR spectral model. Different methods were applied to the selection of response bands interval to construct robust inversion models. Among them, VIP method could select larger number of wavebands with the highest accuracy, SR method could select the smallest number of wavebands with good accuracy. However, the number of wavebands obtained using the GC method varied greatly with poor accuracy. The PLSR and SVR models achieved good effects on the modeling and forecasting of most ions content. Moreover, the PLSR model was slightly more than the SVR model in terms of the number of ion models with good predictive effects (RPD over 2.0). The models of Ca^2+^, Na^+^, Cl^−^, Mg^2+^ and SO_4_^2−^ displayed the highest prediction accuracy, and the RPDs were 3.97, 3.15, 2.98, 2.75 and 2.75, respectively, while those of other ions were poor. Overall, the best wavelength selection methods, models and inversion results of soil salt ions were different. In the future, the combination of band selection methods and spectral model will have a great potential for predicting some soil salt ions content in the salinization area. Such an approach can be utilized to assist decision makers toward the determination of soil salinization levels.

##  Supplemental Information

10.7717/peerj.6310/supp-1 Supplemental Information 1 Spectral datas of reflectance and SNV reflectance

## Additional Information and Declarations

### Field Study Permissions

The following information was supplied relating to field study approvals (i.e., approving body and any reference numbers): The Hetao irrigation district administration approved the field sampling (2017YFC0403302).

### Data Availability

The following information was supplied regarding data availability: The raw data are available in the Supplemental File.
